# An Update on Crop ABA Receptors

**DOI:** 10.3390/plants10061087

**Published:** 2021-05-28

**Authors:** Rafael Ruiz-Partida, Sttefany M. Rosario, Jorge Lozano-Juste

**Affiliations:** 1 Consejo Superior de Investigaciones Científicas (CSIC), Instituto de Biología Molecular y Celular de Plantas (IBMCP), Universitat Politècnica de València (UPV), Calle Ingeniero Fausto Elio s/n, Edificio 8E, 46022 Valencia, Spain; rafarupa@gmail.com (R.R.-P.); mer.rosario@gmail.com (S.M.R.); 2 Laboratorio de Biología Molecular, Facultad de Ciencias Agronómicas y Veterinarias, Universidad Autónoma de Santo Domingo (UASD), Camino de Engombe, Santo Domingo 10904, Dominican Republic

**Keywords:** crop, ABA, receptor, PYR/PYL, structure, phylogenetic, drought

## Abstract

The hormone abscisic acid (ABA) orchestrates the plant stress response and regulates sophisticated metabolic and physiological mechanisms essential for survival in a changing environment. Plant ABA receptors were described more than 10 years ago, and a considerable amount of information is available for the model plant *Arabidopsis thaliana*. Unfortunately, this knowledge is still very limited in crops that hold the key to feeding a growing population. In this review, we summarize genomic, genetic and structural data obtained in crop ABA receptors. We also provide an update on ABA perception in major food crops, highlighting specific and common features of crop ABA receptors.

## 1. Introduction

Abscisic acid (ABA) is a phytohormone involved in the regulation of many aspects of plant development and adaptation to external cues. The first steps of ABA biosynthesis take place in the chloroplast, moving on into the cytosol where the bioactive sesquiterpene (S)-(+)-Abscisic acid is finally synthesized. Under some stress conditions (e.g., drought), plants accumulate large amounts of ABA through the transcriptional modulation of ABA biosynthesis and degradation genes [1,2,3]. Simultaneously, drought stimulates the liberation of bioactive ABA from glucose-conjugated ABA stores by enhancing the transcription of dedicated β-glucosidases [4,5]. Both processes contribute to an elevation of ABA levels that is also coordinated with the transport of the hormone. ABA exits the cells by the action of ABA exporters, AtABCG25 and AtDTX50, located in vascular parenchyma cells and the vegetative-vascular tissue, respectively [6,7,8]. ABA is distributed throughout the plant, moving through the xylem and entering into the cells by import transporters like AtABCG40, located in guard cells and in the embryo, and via the NRT1.2 importer in the vasculature [9,10,11]. In the cell, the PYR/PYL receptors, a family of soluble proteins able to interact and inhibit clade A PP2C phosphatases, binds ABA in their hydrophobic pocket. From here, a chain of molecular events reset the plant strategy from growth and development to risk minimization, contributing to plant adaptation to stress. ABA receptors are at the tipping point of the balance between plant growth and survival.

The identification of a plant ABA receptor was first reported in 2006. Razem et al. proposed that the RNA-binding protein FCA, a well-known regulator of the floral transition, was able to bind ABA and regulate some ABA-dependent responses [12]. However, this work was retracted two years later due to errors in the binding assays and lack of reproducibility, as stated by the authors [13]. Also in 2006, the group of Da-Peng Zhang proposed the H subunit of the Magnesium Chelatase (CHLH) as an ABA receptor (ABAR) [14]. Biochemical, genetic and physiological experiments supported this notion, but other labs were not able to replicate their results, especially those proving that CHLH binds ABA. G protein-coupled receptors have also been proposed as plant ABA receptors [15,16], but reproducibility issues in binding assays or in the phenotypes of the loss-of-function mutants ruled them out [17,18]. However, in 2009, two different groups discovered the pyrabactin resistance (PYR) 1/PYR1-like (PYL)/regulatory component of ABA receptor (RCAR), ABA receptors simultaneously using *Arabidopsis thaliana* (Arabidopsis) as a model plant [19,20]. After the discovery, other groups confirmed their findings and very quickly, a vast amount of information on PYR/PYL ABA receptors was published, all confirming the discovery by these two labs and indicating that previously reported ABA receptors are probably artifacts unlikely to bind ABA. Furthermore, the generation of high-order PYR/PYL mutants, insensitive to ABA, even when using exceptionally high ABA concentrations (i.e., 100 µM), indicates that even if there are other proteins able to bind ABA, their role must be very minor [21,22]. Soon after their discovery, structural biologists were able to solve the crystal structure of the PYR/PYL receptors in complex with ABA and, subsequently, the crystallization of the ternary complex formed by the receptor, ABA and the PP2C [23,24,25,26,27,28,29,30]. Crystal structures provide valuable information about the molecular mechanism of ABA perception and explain the interaction between ABA, the PYR/PYL receptors and the protein phosphatases 2 C (PP2C), at the molecular level, indicating that PP2Cs might act as ABA co-receptors [31]. Under non-stress conditions, the plant ABA levels are low and, consequently, clade A PP2C phosphatases dephosphorylate a subset of SnRK2 kinases important for stress signaling [32]. Under stress conditions, (e.g., drought), ABA levels increase and PYR/PYL receptors bound to ABA interact with and inhibit PP2Cs. The interaction and the inhibition of PP2Cs by PYR/PYL receptors favor the activity of SnRK2s, which phosphorylate and activate transcription factors and membrane channels essential for the adaptation to drought [32].

PYR/PYL proteins are members of the START domain superfamily and have gained their ability to bind ABA during evolution. Algae PYR/PYLs do not bind ABA but in turn, they interact and inhibit PP2Cs. This has been recently discovered using *Zygnema circumcarinatum*, an ancestor of land plants [33]. ZcPYL8, the only PYR/PYL protein encoded in the *Z. circumcarinatum* genome, cannot bind ABA, but is able to interact with and inhibit PP2Cs in a PYL concentration-dependent manner [33]. This suggests that PYR/PYL proteins have an ABA-independent ancestral role and do not require ABA for their function. However, in land plants, PYR/PYL proteins have gained the ability to bind ABA and to regulate PP2C inhibition in a ligand-dependent manner. In Bryophytes like *Marchantia polymorfa* or *Physcomitrella patens*, PYR/PYL proteins have evolved and can bind ABA with K_D_ values similar to those of PYR/PYL receptors of higher plants [33]. However, they still retain the ability to inhibit PP2Cs independently of ABA binding. Lastly, angiosperms have developed a more sophisticated subfamily of PYR/PYL proteins which are able to dimerize in the absence of ABA. In this case, PYR/PYLs use their PP2C interaction surface as a docking platform for homodimerization, which prevents the interaction with PP2Cs and, consequently, their inhibition. The binding of ABA induces a conformational rearrangement, which results in the dissociation of the PYR/PYL dimer allowing for the interaction with the PP2C. This molecular strategy limits the inhibition of PP2Cs to ABA-bound PYR/PYLs exclusively, repurposing the original ligand-independent regulatory role of ancestral PYLs into a ligand-binding dependent process [34].

The ability of PYR/PYL proteins to bind ABA has been a key sophistication acquired during plant evolution [34,35]. It allows plants to finely regulate transpiration, water use and stress tolerance, thus providing a mechanism to expand their habitat from aquatic settings to dry land environments. ABA receptors are therefore key targets to manipulate plant transpiration, stomata closure and stress adaptation. Since the discovery of PYR/PYL proteins, major efforts have been made to capitalize on this knowledge to develop biotechnological strategies to cope with water stress [36,37,38,39]. The reduction in precipitations and fresh water availability imposed by climate change has urged researchers to find solutions to secure a sufficient food supply for a growing population. Different strategies have been developed, based on genetic or chemical manipulation of PYR/PYL activity. However, despite the relevance of crop plants in this context, most of the tools have been generated in the model plant Arabidopsis, which is more suited to genetic and chemical intervention. Nonetheless, recent findings using non-model organisms point to relevant differences between Arabidopsis PYR/PYLs and ABA receptors from other plant species. In this review, we discuss recent developments in crop ABA receptors, with a special focus on staple food crops.

## 2. ABA Receptors in Major Food Crops

Based on their commercial use, crops can be classified into food crops (e.g., rice), cash crops (plants that are commercialized into non-food products, e.g., sugarcane), forage crops (used to feed grazing animals, e.g., annual warm-season grasses) and food adjuncts (including spices, coffee, tea, etc.). In this review, we focus our attention on food crops, and among them, on the three most important monocot crops (maize, wheat and rice) and another three eudicot food crops (soybean, tomato and citrus). We have selected these species based on their cultivation and productivity, and the available information on ABA receptors in these species. It is interesting to mention that from more than 100,000 known plant species, we cultivate and exploit only around 120. Nine of them account for 75% of the calories that humans consume from plants. Interestingly, maize, wheat and rice provide more than 50% of them [40].

According to 2019/2020 FAOSTAT data [40] wheat (*Triticum aestivum*, Ta) is the most cultivated food crop worldwide, with an extension of 217 million hectares (MH), followed by maize (*Zea mays*, Zm), with 193 MH and rice (*Oryza sativa*, Os), with 160 MH. For eudicot crops, soybeans (*Glycine max*, Gm) are the most cultivated, with 122 MH ([40]. However, in terms of productivity, the figures are quite different. Maize production in 2019/2020 was about 1148 million tons (Mt), while wheat production reached 765 Mt, and rice 755 Mt. Eudicot crop productivity is lower than monocots, but very important. Potato, soybean and cassava are the most highly produced eudicot food crops, with 370 Mt of potato, 333 Mt of soybeans and 303 Mt of cassava produced last year [40]. Moreover, we found no information on potato or cassava ABA receptors in the literature, exemplifying the urgent need to extend the findings developed in model plants to relevant food crops. For this review, we have focused on soybean, tomato (*Solanum lycopersicum*, Sl) and citrus (*Citrus sinensis*, Cs), as they are the next highest producing eudicot plant crop species from which data on PYR/PYL receptors have been reported. Tomato is the fourth eudicot crop in production with 180 Mt and citrus is the fifth with 78 Mt of fruit produced per year [40]. It is relevant to mention that sugarcane is actually the most highly produced crop in the world. However, it is transformed and sold as sugar, alcohol or ethanol for biofuels. It is therefore considered a cash crop rather than a food crop. Unfortunately, we have not been able to find reports on sugarcane ABA receptors.

**Figure 1:**
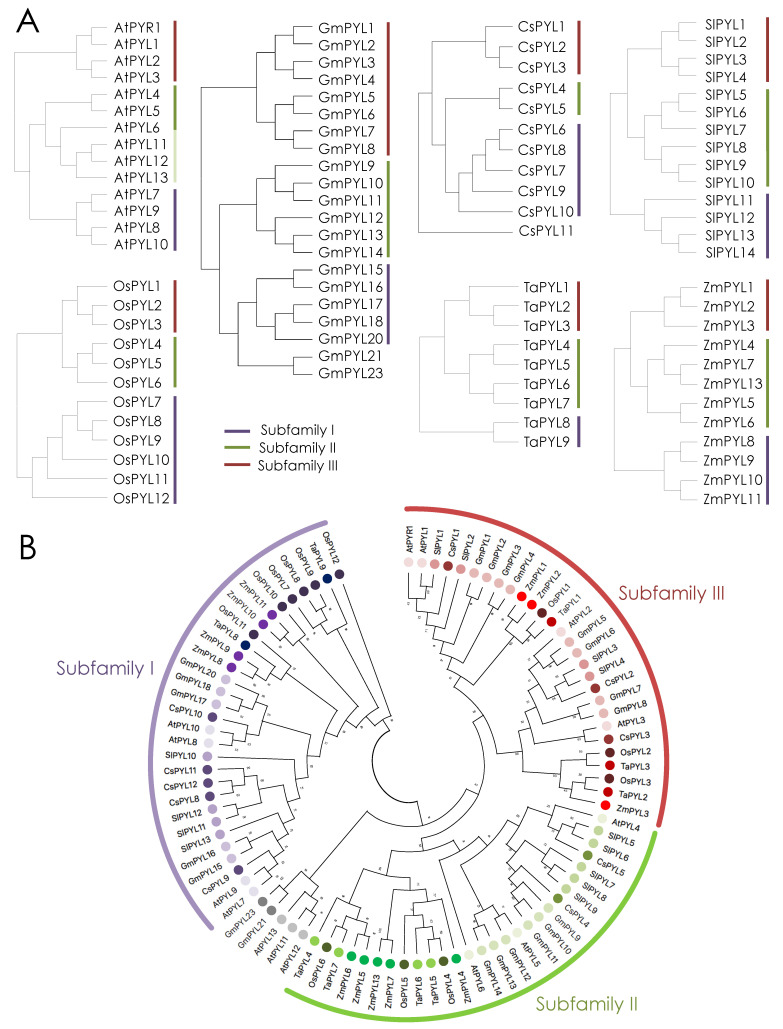
ABA receptors in different crops. Cladograms (**A**) and circular phylogenetic tree (**B**) of PYR/PYLs in eudicot and monocot food crops. Receptors are grouped in three different subfamilies: subfamily I (purple), subfamily II (green) and subfamily III (red). Cladograms and the circular phylogenetic tree were built with the maximum likelihood method. The phylogenetic tree was built using 1000 bootstraps in MEGA. Numbers indicate bootstrap values.

PYR/PYL ABA receptors have been identified in the major food crops maize, rice, wheat, soybean, tomato and citrus and present slightly different gene family sizes (Figure 1) [39,41,42,43,44,45,46,47,48,49]. In tomato, ABA receptors form a family of 14 members, the same number of receptors that have been found in the Arabidopsis eudicot model (Figure 1A) [45,50]. Eleven receptors have been found in *Citrus sinensis* (Figure 1A) [46,47]. The situation is different in soybean, which is a partially diploidized tetraploid that contains 23 ABA receptors (Figure 1A) [41]. In monocots, the family of ABA receptors is similar or slightly reduced. The maize genome contains 12 ABA receptors [48,49], the same number of receptors found in rice, while 9 types of ABA receptors have been described in wheat (Figure 1A) [39,44]. Similarly, the C3 monocot model *Brachypodium distachyon* contains 9 functional ABA receptors and the C4 monocot model *Setaria viridis* encodes for 8 ABA receptors in contrast with the 14 receptors found in Arabidopsis and tomato [51,52].

Despite the identification of ABA receptors in these economically relevant species, biochemical data proving ABA binding is lacking for many of them. PP2C phosphatase inhibition assays, where PYR/PYL proteins are able to inhibit PP2C activity only in the presence of ABA, are indirect, but convenient and robust indicators for ABA binding. Only receptors in wheat, rice and some of the tomato, citrus and soybean have been analyzed in vitro in PP2C assays [39,41,45,46,53]. For soybean receptors, protein–protein interaction assays in heterologous systems have been reported. GmPYL proteins and the PP2C phosphatase AtABI1 interact in vivo in yeast cells and this interaction occurs both in the presence and in the absence of ABA [41]. The soybean receptor GmPYL1 and AtABI1 are also able to interact in epidermal cells of *Nicotiana benthamiana* after infiltrating the leaves with agrobacterium in the presence of ABA in BiFC experiments [41]. Soybean receptors have also been studied in vitro. Using a high receptor-phosphatase ratio (5:1), 3 out of the 23 soybean receptors, GmPYL1, GmPYL16 and GmPYL21, were shown to be active and inhibit PP2C activity only in the presence of ABA [41]. The information about the in vitro activity of reported ABA receptors is also partial in tomato. In vitro activity data is reported only for 5 out of 14 tomato receptors. In the case of maize, we could not find any reported in vitro activity for the proposed maize ABA receptors. Fortunately, the biochemical characterization of PYR/PYLs in rice and wheat has been addressed [39,44]. The ability to bind ABA of all wheat and rice ABA receptors has been tested in PP2C assays and even the dissociation constants (K_D_) for certain rice receptors have been calculated using Isothermal Titration Calorimetry (ITC) [39,44].

ABA receptors cluster in three different subfamilies (I, II and III) conserved among angiosperms (Figure 1B) [34]. These subfamilies have arisen during evolution and subfamily III has been the latest to emerge and is present only in angiosperms. Subfamily I and II are composed of monomeric receptors and the receptors of subfamily III adopt a dimeric configuration [25,29].

Based on Arabidopsis data, monomeric receptors have higher affinity for ABA than dimeric receptors [29,38]. This is explained by the requirement of ABA to induce the dissociation of the homodimers from subfamily III receptors, prior to the interaction with the PP2Cs, and by the presence of certain residues in monomeric receptors [29]. There are few reports on the direct measurement of ABA binding by PYR/PYL receptors, and almost all of them have been conducted using Arabidopsis receptors. Dimeric receptors AtPYR1, AtPYL1 and AtPYL2 have low affinity for ABA and therefore a high dissociation constant (K_D_ > 50 µM, 52 µM and 59 µM, respectively) [29]. However, the affinity for ABA considerably increases (and therefore, the constant drops) when the PP2C phosphatase is included in the assay, probably due to a reduction in the dissociation rate of ABA in the ternary complex [29,54]. For example, AtPYR1 exhibits a K_D_ for ABA of 97 +/− 36 µM when measured using NMR [29]. Interestingly, this affinity significantly increases, showing a K_D_ value of 200 nM, when the PP2C phosphatase is included in the ITC assay. In contrast, the monomeric AtPYL5 has a dissociation constant of 1 µM in the absence of PP2C, more than 50 times lower than that reported for dimeric receptors. Again, the introduction of the PP2C AtHAB1 in the ITC assay increases the affinity of AtPYL5-AtHAB1 for ABA to 38 nM [38]. 

In crop ABA receptors, the available information on the direct measurement of ABA binding for PYR/PYL receptors is very scant. Only the dimeric rice OsPYL2 receptor has been analyzed. It has been found that the K_D_ of OsPYL2 for ABA is very similar to that of Arabidopsis dimeric receptors. Using ITC experiments, the K_D_ for OsPYL2 was estimated to be 31 µM, indicating that the low affinity for ABA of dimeric receptors is conserved between rice and Arabidopsis [44]. Unfortunately, the K_D_ of dimeric or monomeric crop ABA receptors has not been studied in the presence of a PP2C. As an alternative, IC50 values for different receptors using PP2C phosphatase assays have been reported. Although PP2C in vitro assays do not directly measure the affinity between the receptor and ABA, there is a relationship between the IC50 and the K_D_ values. For example, while AtPYR1 and AtPYL1 receptors have an IC50 for ABA in PP2C assays of 300 nM, monomeric AtPYL5 exhibits an IC50 value of 27 nM [55]. Accordingly, the K_D_ of AtPYL1 is 52 µM while AtPYL5 has a K_D_ of 1 µM [29,38]. Actually, all Arabidopsis monomeric receptors show IC50 values lower than 70 nM, with the exception of AtPYL10 with an IC50 of 121 nM. In contrast, all dimeric receptors’ IC50 values are higher than 150 nM, with AtPYR1 and AtPYL1 showing an IC50 value higher than 300 nM [55]. However, IC50 data reported for wheat receptors show different results [39]. There is not a clear difference between dimeric and monomeric IC50 values. The dimeric receptors TaPYL1 and TaPYL2 have IC50 values of 239 and 81 nM, respectively [39]. Monomeric receptors, TaPYL7 and TaPYL8 show similar IC50 values of 95 nM and 328 nM, respectively [39]. Other monomeric receptors like TaPYL5 show IC50 values of 43 nM [39]. This indicates that there might be differences among plant species, and suggests that the assumption of dimeric and monomeric isoforms having different affinity for ABA might need to be carefully investigated. In any case, this observation requires further validation since the difference in IC50 values could be due to the use of different protein tags or slightly different enzymatic assay conditions. The direct measurement of the dissociation constants (e.g., ITC) would be necessary to properly address this point.

In Arabidopsis, subfamily II includes AtPYL4, AtPYL5 and AtPYL6, in one clade, and AtPYL11, AtPYL12 and AtPYL13 forming another [55]. Interestingly, the clade formed by AtPYL11/12/13 is missing in all the monocot species analyzed here (Figure 1). We have also looked for this group in other non-crop monocots (*Brachypodium distachyon* and *Setaria viridis*) with the same result. Interestingly, in tomato and citrus the AtPYL11/12/13 group is also missing (Figure 1). In the case of the soybean genome, which encodes for 23 ABA receptors, it is possible that GmPYL21/22/23 mirror AtPYL11/12/13 (Figure 1). Although more information on other plant species is needed, it seems that AtPYL11/12/13, or Gm21/22/23, are restricted to only some plant species and have not been conserved among the plant lineage. Actually, the expression levels of these genes are very low in Arabidopsis RNAseq datasets, indicating that either they have a very specific role in a restricted tissue or cell type, or that they have rather a minor role. Indeed, genetic combination of pyl11/12 with other low expressed *PYL*s in a pentuple mutant, *pyl3/7/9/11/12*, results in wt sensitivity to ABA, as opposed to the strong ABA insensitivity of the *pyl1/2/4/5* mutant [21,22]. Also, in soybean, the expression levels of these genes are very low and *GmPYL23* was found to be transcriptionally inactive among 14 RNAseq experiments (SoyBase; https://soybase.org accessed on 15 March 2021).

Subfamily I of ABA receptors, also known as the AtPYL8-like subfamily, is believed to include important receptors for root development and hydrotropism [56]. Overall, all crops analyzed in this work show a similar number of ABA receptors in this subfamily I. One notable exception is wheat, which shows a severe reduction in AtPYL8-like receptors, counting only two types of subfamily I receptors, while rice or citrus have six or five receptors in this subfamily, respectively (Figure 1). Whether wheat has a specific requirement for root growth or other wheat PYL receptors have taken over the role of AtPYL8-like receptors requires further experimentation. Reported data indicate that AtPYL8 is a singular ABA receptor. Loss-of-function of AtPYL8 leads to a phenotype of reduced sensitivity to ABA in root growth [56,57]. This is one of the very few examples where a single mutant in one of the 14 Arabidopsis PYR/PYL receptors leads to an observable phenotype. Thus, AtPYL8 has a specific role in roots. Additionally, while it is known that some ABA receptors are subjected to ubiquitination and degradation in response to ABA, AtPYL8, in contrast, is not degraded but stabilized by ABA [57,58,59,60,61,62,63,64]. AtPYL8 accumulates in the presence of ABA in the plant nuclei through a mechanism that relies on hormone perception since a double AtPYL8^K61A,Y120A^ mutant receptor, unable to bind ABA, is not stabilized [57]. Moreover, the AtPYL8 protein is able to move from the epidermal cells of roots into more internal root cell layers, suggesting that AtPYL8 can expand its action to the cortex, endodermis or even the vasculature when ABA levels are high [57]. Among crops, the stability of PYL8 has only been studied in date palm receptors (*Phoenix dactilyfera*) [65]. PdPYL8/Pd27 is accumulated in response to ABA treatment in transgenic Arabidopsis overexpressing PdPYL8/Pd27, as occurs with AtPYL8 [65]. These results suggest that the ABA-dependent stabilization of PYL8 might be conserved among PYL8-like receptors of different plant species. Whether this mechanism is also conserved in other food crops still needs to be characterized. 

## 3. Genetic Characterization of ABA Receptors in Crops

*Arabidopsis thaliana* is an exceptional model system to study plant genetics. Many mutants in PYR/PYL receptors have been obtained in Arabidopsis. During a forward chemical genetic screening, 10 alleles of the *PYR1* gene were identified (*pyr1-1* to *pyr1-10*) by their resistance to the ABA-receptor agonist pyrabactin [19]. Using public T-DNA collections, the quadruple mutant, *pyr1pyl1pyl2pyl4* (QC3), including the *pyr1-1* allele, was generated. *pyr1pyl1pyl2pyl4* has an important ABA insensitivity phenotype in seed germination [19]. While wt seed cannot germinate in the presence of 1 µM ABA and root growth is reduced by 50% by 10 µM ABA, the quadruple mutant is able to germinate, to produce green cotyledons and the root growth of this mutant is not impaired at 10 µM ABA [19]. This important insensitivity phenotype could seem minor when compared to the extreme ABA insensitivity shown by the *pyr1pyl1pyl2pyl4pyl5pyl8* (*112458*) sextuple mutant [22]. The *112458* sextuple mutant was generated by crossing and selecting different combinations of pyr/pyl T-DNA lines. *112458* can germinate and shows normal root growth in the presence of 100 µM ABA, indicating the importance and the redundancy of these receptors in ABA perception and signaling [22]. Furthermore, a quattuordecuple (14) mutant in all Arabidopsis ABA receptors has been generated using genome editing of the *112458* mutant [21]. This quattuordecuple mutant is not fertile, probably due to male sterility, and presents severely impaired growth [21], although the off-target effects of the 8 sgRNAs used to generate the mutant have not been reported. Researchers have also generated a duodecuple (12) mutant, *pyr1pyl123456789,10,11* (*112458 36791011*), which is, in this case, fertile. The ABA insensitivity of this mutant is slightly higher than that observed in the sextuple *112458* mutant, indicating a rather minor role for some receptors (AtPYL12 and AtPYL13) in the regulation of ABA-dependent seed germination, root growth, stomata closure and gene expression. In germination assays, *112458* seeds can germinate in 100 µM ABA, but their germination is reduced when 200 µM is used. However, the *112458 36791011* mutant germinates perfectly under 200 µM ABA [21]. A similar situation is observed when root growth, stomata closure or gene expression are analyzed in *112458 36791011* mutants, which show a slightly more pronounced insensitivity to exceptionally high ABA concentrations [21]. These phenotypes also indicate a minor role for the AtPYL12 and AtPYL13 ABA receptors.

The genetic analysis of ABA receptors in crops is very far behind that performed in Arabidopsis. There are no knock-out mutants reported for any ABA receptor in wheat, maize, soybean, tomato or citrus (Table 1). An effort towards the genetic characterization of crop ABA receptors is thus needed to capitalize on PYR/PYL receptors to improve crop yield under stress conditions. Fortunately, extensive work has been carried out in rice to characterize OsPYLs (Table 1) [53]. In their work, the authors identify ABA receptors important for stomatal movement, seed germination and growth regulation in rice [53]. By using CRISPR/Cas9, Miao and co-workers generated a battery of rice mutants combining different ABA receptors. Loss-of-function of subfamily II and III receptors led to insensitivity to ABA in seed germination. The *Ospyl1/2/3/4/5/6/12* mutant is able to germinate in the presence of 10 µM ABA while wt seeds cannot germinate in 5 µM ABA [53]. Interestingly, mutants in subfamily I, like *Ospyl7/8/9/10/11/13*, were as sensitive to ABA as the wt in seed germination, indicating that the role of PYL subfamily I receptors in rice germination is minor [53]. The *Ospyl1/2/3/4/5/6/12* mutant also showed a higher index of preharvest sprouting, in agreement with their insensitivity to ABA. However, this is not a desirable trait, since it allows the germination of grains in the panicle, ruining rice yield. Stomata closure was also affected in these mutants. In this case, the *Ospyl1/2/3/4/5/6/12* mutant is affected in ABA-induced stomatal closure, while *Ospyl7/8/9/10/11/13* did not show an obvious phenotype [53]. This data indicates that subfamilies II and III are important for drought tolerance in rice. Strikingly, mutants in subfamilies II and III grow faster than wt plants under certain growth conditions. Specifically, *Ospyl1/4/6* plants are taller with longer and more branched panicles than wt, which results in an increased biomass in *Ospyl1/4/6 plants*. Indeed, under paddy field conditions, *Ospyl1/4/6* produces 25–31% more grain without showing any preharvest sprouting issues [53]. However, under drought stress conditions, *Ospyl1/4/6* plants behave worse than wt plants. It is therefore clear that OsPYL1, OsPYL4 and OsPYL6 have important roles in rice and are promising targets for crop improvement. 

Although there are no reports on knock-out mutants for the PYR/PYL receptors in wheat, maize, soybean, tomato and citrus in the literature, there are several examples of the generation of gain-of-function and loss-of-function transgenic plants. Using this approach, PYR/PYL overexpression has been shown to lead to ABA hypersensitivity and drought tolerance. For example, overexpression of OsPYL3, OsPYL5, OsPYL9 and OsPYL11 in transgenic rice improves performance under drought conditions (Table 1) [43,66,67]. This is explained by the higher sensitivity to ABA of these transgenic plants, as shown in germination assays and gene expression analysis. However, this increase in stress tolerance comes at a cost to growth and grain production. For instance, rice plants overexpressing OsPYL5 grow well under controlled settings, but under paddy field conditions, the transgenic lines show a reduction in height due to reduced internode length and leaf length, and importantly a 66% reduction in grain weight [67]. In addition, the overexpression of SlPYL9 in Microtom tomato leads to increased sensitivity to ABA and better performance under drought conditions, compared to wt plants (Table 1) [50]. Interestingly, SlPYL9 seems to have a specific role in fruit development and ripening. SlPYL9 overexpression plants show accelerated fruit ripening compared to the wt, while SlPYL9-RNAi lines show a delay in ripening [50]. Misexpression of SlPYL9 also leads to changes in fruit quality. Fruit firmness and fruit shape index are reduced in SlPYL9 overexpressing plants, while they are increased in SlPYL9-RNAi lines [50]. However, the overexpression of SlPYL9 also produces an undesired reduction in total fresh weight, indicating that PYR/PYL overexpression in tomato under a constitutive promoter could have negative implications for yield [50]. The negative effect of PYL overexpression can be bypassed by using inducible promoters or engineered receptors, as exemplified in the *pRD29a-AtPYL2* transgenic soybean and the *35S:PYR1^MANDI^* tomato [68,69] (Table 1). RD29a promoter is highly activated by ABA and drought conditions [70,71].

**Table 1:** Reported transgenic crops with altered PYL expression. The gene code, transgenic effect, transgenic plant, phenotype and reference are indicated.

No.	Gene	Effect	Transgenic Plant	Phenotype	Reference
**1**	OsPYL1/2/3/4/5/6/12	Knock-out (CRISPR/Cas9)	Rice	ABA insensitivity, increased productivity under paddy field conditions, preharvest sprouting	Miao et al., 2018 [53]
**2**	OsPYL7/8/9/10/11/13	Knock-out (CRISPR/Cas9)	Rice	No obvious phenotype	Miao et al., 2018 [53]
**3**	OsPYL1/4/6	Knock-out (CRISPR/Cas9)	Rice	ABA insensitivity, increased productivity under paddy field conditions	Miao et al., 2018 [53]
**4**	OsPYL3, OsPYL9	Overexpression	Rice	ABA hypersensitivity, drought resistance	Tian et al., 2015 [43]
**5**	OsPYL5	Overexpression	Rice	ABA hypersensitivity, drought and salt resistance.	Kim et al., 2014 [67]
**6**	OsPYL11	Overexpression	Rice	ABA hypersensitivity	Kim et al., 2012 [66]
**7**	SlPYL9	Overexpression	Tomato	ABA hypersensitivity, accelerated fruit ripening	Kai et al., 2019 [50]
**8**	SlPYL9	Knock-down (RNAi)	Tomato	ABA hyposensitivity, delayed fruit ripening	Kai et al., 2019 [50]
**9**	AtPYR1^MANDI^	Overexpression	Tomato	ABA hypersensitivity and drought resistance in response to the agrochemical Mandipropamid	Park et al., 2015 [69]
**10**	AtPYL2	Inducible expression	Soybean	Drought resistance	Cao et al., 2017 [68]
**11**	TaPYL4	Overexpression	Wheat	ABA hypersensitivity, increased water productivity, drought resistance	Mega et al., 2019 [39]

Soybean plants (Ws82 wild-type) do not survive water withdrawal for seven days in the conditions reported by Cao and co-workers, showing around 10% survival at day seven and 0% survival at day eight after water deprivation. However, *pRD29a-AtPYL2* transgenic soybean lines retain 100% survival at day seven and 60% of the plants survived eight days of no irrigation [68]. The drought resistance of *pRD29a-AtPYL2* transgenic soybean plants seems even stronger than that of Ws82 wt plants treated with ABA, indicating a strong drought resistance effect of the conditional expression of the AtPYL2 receptor [68]. Notably, *pRD29a-AtPYL2* transgenic soybean plants do not show any growth or developmental defects compared to Ws82 wt plants, thus validating the use of inducible promoters to reduce yield drag associated with PYR/PYL overexpression. The development of engineered ABA receptors that are selectively activated by small molecules is also a promising strategy that minimizes the negative impact of PYR/PYL overexpression on crop yield, and allows to activate the ABA signaling pathway on demand [69]. Surprisingly, there are data indicating that reduction in yield due to ABA-receptor overexpression could be species- or receptor-specific. In an elegant study, overexpression of TaPYL4 (TaPYL4ox) in wheat was shown to improve drought tolerance without a negative impact on plant yield (Table 1) [39]. Moreover, TaPYL4ox lines have higher water-use efficiency (WUE), not only under water-limiting conditions, but also under well-watered conditions [39]. TaPYL4ox seeds are more sensitive to ABA in germination and seedling growth assays. This increased sensitivity to ABA is translated into reduced water loss and stomata apertures, eventually showing reduced transpiration compared to the wt. Under water-limiting conditions, higher water use efficiency (WUE) observed for transgenic TaPYL4ox wheat plants is partly due to an increase in photosynthetic capacity, while keeping stomata apertures reduced compared to wt plants [39]. Importantly, these features are translated into higher grain production under water-limiting conditions. Wheat grain yield is severely affected by drought, producing smaller and shrunken grains [39]. However, TaPYL4ox grains are normal and their size is only slightly reduced by drought. Furthermore, grain quality in TaPYL4ox plants is not affected, and it is even better than wt grains produced under water stress conditions [39]. Taken altogether, this work indicates that TaPYL4 overexpression in wheat improves water use efficiency without any phenotypic or yield penalty. This is an example of a plant species where the increased sensitivity to ABA does not have a negative effect on plant performance as opposed to what has been reported in many cases, such as Arabidopsis and rice. 

An effort to unveil crop PYR/PYL genetics is needed to determine their role in plant growth and productivity, and to identify the best targets to improve stress tolerance in crops.

## 4. Gene Expression of PYR/PYL Receptors in Crops

As commented above, ABA receptors are interesting targets to improve plant resistance to drought. However, without genetic data indicating the function of each PYL in crops, it is difficult to identify the most appropriate receptor to use in breeding strategies. Alternatively, the analysis of the expression patterns of the different crop *PYR/PYL* genes could help in the selection of the best candidates. 

It is widely accepted that ABA reduces the expression of the *PYR/PYL* receptors and induces the expression of the *PP2C* phosphatases to avoid a sustained ABA response that could be detrimental for plant performance. While this is true for many Arabidopsis receptors, expression analyses on different plant species suggest a different scenario. In Arabidopsis, *AtPYR1*, *AtPYL1*, *AtPYL4*, *AtPYL5*, *AtPYL6* and *AtPYL8* are strongly down-regulated by ABA [38,72,73], while the other receptors show little changes in expression. In citrus, this tendency is conserved, but only *CsPYL2*, *CsPYL4* and *CsPYL5* are down-regulated in situations where ABA levels are high [46]. Interestingly, under the same conditions, citrus *CsPYL8* is up-regulated, in contrast to Arabidopsis *AtPYL8*. Accordingly, *CsPYL8* and *CsPYL9* expression is down-regulated when ABA levels are low [46]. Soybean *PYR/PYL* genes also show a mixed regulation by ABA: *GmPYL3*, *GmPYL5*, *GmPYL7*, *GmPYL8*, *GmPYL9*, *GmPYL11*, *GmPYL14*, and *GmPYL15* are down-regulated by ABA but *GmPYL1*, *GmPYL10*, *GmPYL12*, *GmPYL13*, and *GmPYL17* are up-regulated after ABA treatment [41]. Tomato *PYLs* do not show important changes—from 0.55 to 1.43-fold—in expression, 24 h after treating plants with 7.5 µM ABA [74]. In rice, among the 12 ABA-receptors identified, *OsPYL4*, *OsPYL5*, *OsPYL6* and *OsPYL10* are down-regulated by ABA while *OsPYL2*, *OsPYL3*, *OsPYL8* and *OsPYL10* are up-regulated by ABA [42]. Intriguingly, in wheat, only *TaPYL1* and *TaPYL4* are repressed in response to ABA, while the expression of the other PYLs remain unchanged [39]. In maize, the situation is very interesting. It has been reported that ABA reduces the expression of dimeric (subfamily III) ABA receptors and induces the expression of monomeric receptors (subfamily I and II) in shoots. However, in roots, the regulation is the opposite; ABA induces dimeric PYL gene expression, while it reduces the expression of monomeric receptors [75]. Altogether, we would like to highlight here that there is not a common trend between ABA treatment and *PYR/PYL* gene expression when several crop species are analyzed. The notion that ABA reduces the expression of PYR/PYLs holds true for most of the Arabidopsis ABA receptors, but this effect is not so clear in crops.

**Figure 2:**
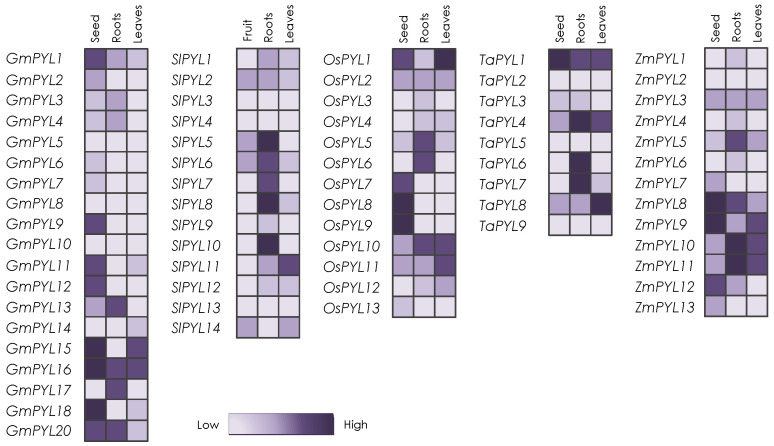
Gene expression of the ABA receptors in different crops. The expression levels of the different genes are represented with increasing purple intensities as indicated in the legend. Expression data were extracted from the references indicated in the text [39,41,42,45,46,49,74].

Regarding questions such as the tissue-specific expression of *PYL* in crops, several reports have been published during recent years. In soybean, monomeric subfamily I genes are the most highly expressed, followed by subfamily II and lastly subfamily III (Figure 2) [41]. Interestingly, the expression is very reduced for subfamily III *GmPYL5-8*, while *GmPYL1-4* are well expressed. In seed tissues, *GmPYL15*, *GmPYL16* and *GmPYL18* show the highest expression. However, in roots, *GmPYL13*, *GmPYL16*, *GmPYL17* and *GmPYL20* show the highest values. In leaves, *GmPYL15* and *GmPYL16* (subfamily I) are the most expressed receptors (Figure 2), indicating that these could be good targets in soybean [41]. For citrus, we have found less information, but one study indicates that the highest expression is for *CsPYL9*, followed by *CsPYL4* and *CsPYL8* [46]. In tomato roots, the subfamily II members, *Solyc10g085310/SlPYL8*, *Solyc03g095780/SlPYL5* and *Solyc10g076410/SlPYL10* are expressed at the highest levels among *PYL*s (Figure 2) [45]. In turn, in leaves, subfamily I *Soly01g095700/SlPYL11* seems to be the gene with the highest expression. In fruits at the breaker stage, subfamily I *Solyc08g082180/SlPYL14*, subfamily II *Solyc03g095780/SlPYL5* and *Solyc06g050500/SlPYL6* along with family III *Solyc06g061180/SlPYL2* are the most expressed genes (Figure 2) [45]. In rice, *OsPYL1* and *OsPYL10* are the most expressed receptors among different tissues. *OsPYL1*, *OsPYL7*, *OsPYL8* and *OsPYL9* have high expression in seed, *OsPYL1*, *OsPYL10* and *OsPYL11* in leaves and *OsPYL5*, *OsPYL6* and *OsPYL10* in roots (Figure 2) [42]. In maize seeds, subfamily I *ZmPYL8*, *ZmPYL9*, *ZmPYL10* and *ZmPYL11* are the most expressed genes. In leaves, subfamily I *ZmPYL9*, *ZmPYL10* and *ZmPYL11* show the highest expression levels, while *ZmPYL10* and *ZmPYL11* are the most highly expressed in roots, followed by *ZmPYL5* (Figure 2) [49,75]. Lastly, in wheat seeds, *TaPYL1* and *TaPYL4* are highly expressed and *TaPYL6* is also well-expressed after seed imbibition [39]. In roots, *TaPYL4*, *TaPYL6* and *TaPYL7* are the predominant receptors and *TaPYL1*, *TaPYL4* and *TaPYL8* are the most expressed genes in leaves (Figure 2) [39].

## 5. Structural Biology on Crop ABA Receptors

Since their discovery in 2009, more than 60 structures of PYR/PYL receptors have been deposited in the Protein Data Bank (PDB). Despite the large number of ABA-receptor structures solved, more than 50 structures correspond to dimeric receptors (subfamily III) and only 7 belong to plant species different from the eudicot model, Arabidopsis. Therefore, there is a lack of structural information on crop ABA receptors as well as on monomeric PYLs. At first glance, due to the high sequence-structure similarity of the PYR/PYL proteins, especially in the ABA binding pocket, the lack of information on crop ABA receptors might not be an important issue. However, there are differences in certain in vitro properties of PYR/PYL receptors among different species, such as their oligomeric state or their PP2C-inhibition activity [31,39,76,77,78]. This question takes on special importance in the design of ABA-receptor agonists/antagonists, given that certain ligands could work in the model plant Arabidopsis, but not in a specific crop.

**Figure 3:**
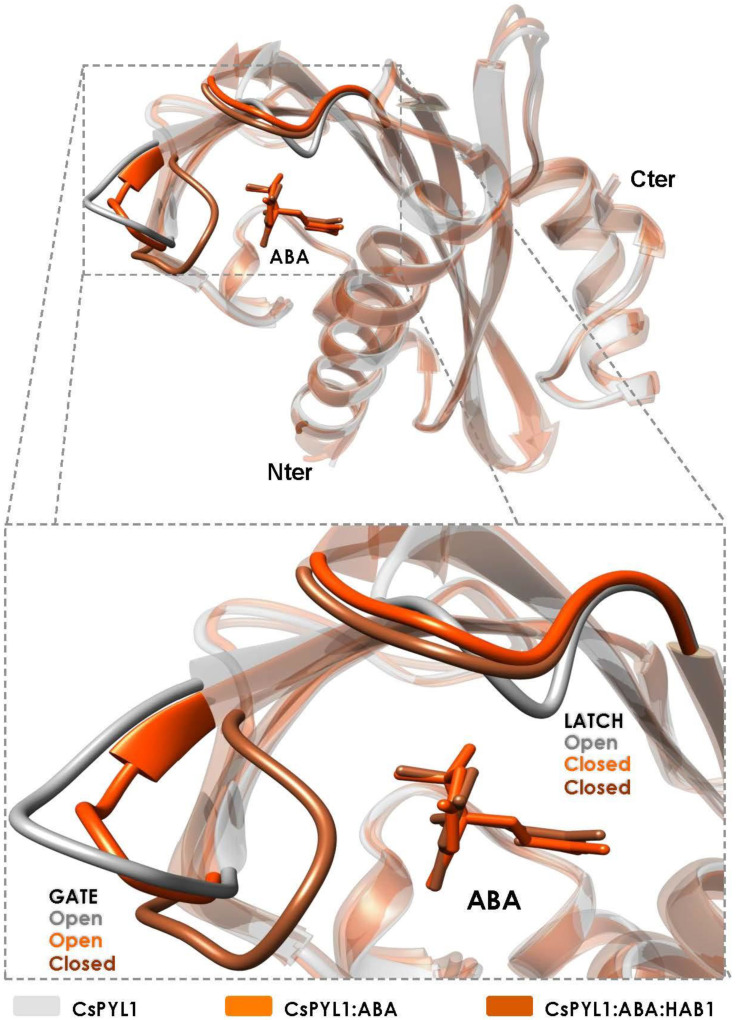
Conformational states of a crop ABA receptor. Superimposition of the CsPYL1 crystal structures corresponding to the apo (PDB ID: 5MMQ), ABA-bound (PDB ID: 5MMX) and ternary complex (PDB ID: 5MN0) forms. The highly conserved latch and gate are highlighted in solid colors.

**Figure 4:**
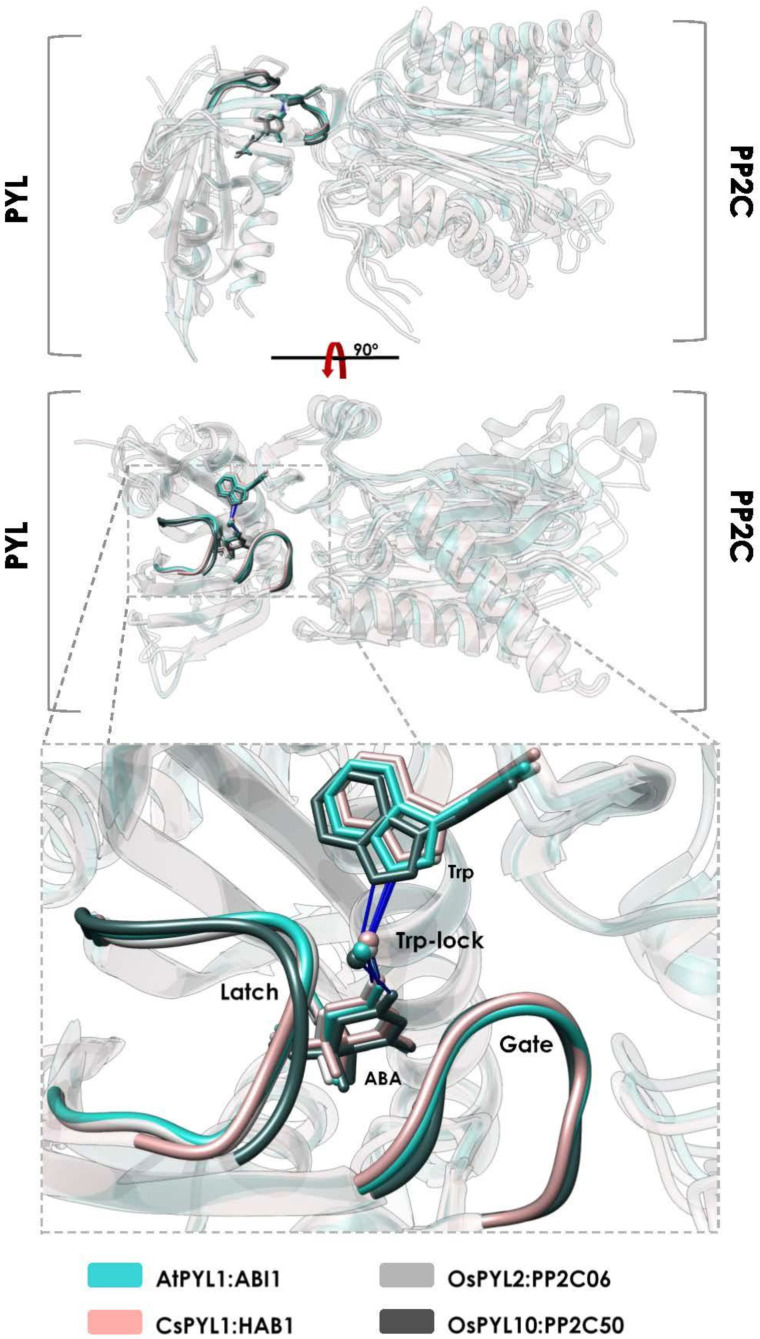
Arabidopsis and crop ABA-receptors adopt a similar latch-closed/gate-closed conformation upon PP2C binding. Structural alignment of PYL:ABA:PP2C ternary complexes from *Arabidopsis thaliana* (PDB ID: 3KDJ), *Citrus sinensis* (PDB ID: 5MN0) and *Oryza sativa* (PDB ID: 4OIC and 5GWP). The bottom panel shows a zoomed-in view of the PP2C tryptophan-lock highlighted in solid colors, similar to the gate and the latch loops.

PYR/PYL proteins adopt an α/β helix-grip fold consisting of seven antiparallel β-strands juxtaposed by a long C-terminal α-helix forming a large hydrophobic cavity, bottom-sealed by two small helices (Figure 3). ABA accommodates in this cavity through a network of water-mediated hydrogen bonds and other electrophilic and hydrophobic interactions [23,27]. The carboxylate moiety of ABA is stabilized through a salt-bridge with a lysine that is conserved in all ABA-receptors analyzed here, except AtPYL13 in Arabidopsis and OsPYL12 in rice [20,23,42,43]. In the ternary complex with clade A PP2C phosphatases, a tryptophan from the PP2C interacts with the ketone moiety of ABA through a water-mediated network of hydrogen bonds known as a tryptophan-lock (Figure 4) [55,79,80]. Therefore, there is a water-mediated contact of the PP2C with the ABA molecule supporting the notion that PP2C phosphatases act as ABA co-receptors (Figure 4) [27,28,81,82]. This tryptophan is conserved among all Arabidopsis clade A PP2Cs, with the exception of AHG1 [28,81,83,84]. Orthologs of AHG1 in other plant species also lack this key tryptophan, resulting in a clade A PP2C that is not regulated by the PYR/PYL receptors [65,85]. Indeed, it has been proposed that the AHG1 ortholog in parasitic Striga prevents ABA signaling, increasing its transpiration under drought conditions to facilitate the absorption of host nutrients, highlighting the particular survival strategy of Striga [86]. 

The ABA-binding cavity of the PYR/PYL receptors is covered with two highly conserved loops (named latch and gate), which act as a lid enclosing ABA in the cavity (Figure 3 and Figure 4) [23,27]. In fact, conformational dynamics of these loops are essential for ABA binding and the interaction with PP2Cs. Upon ABA binding, the loops adopt a closed conformation generating a solvent-exposed surface complementary to the active site of the PP2C (Figure 3 and Figure 4) [27,28,31]. Crystal structures of the apo forms of the Arabidopsis receptors AtPYR1, AtPYL1 and AtPYL2 show a conformation in which the latch and the gate are in an open conformation, in contrast to AtPYL3 and AtPYL10 in which the latch adopts a closed conformation [31]. This indicates that the apo forms of ABA receptors are in a conformational equilibrium between latch-open/gate-open and latch-closed/gate-open conformations. On the other hand, structures of Arabidopsis ABA-bound receptors, as well as the ternary complexes with PP2Cs, exhibited a receptor conformation in which the latch and the gate are in a closed conformation [23,27]. In addition, two tunnels adjacent to 3′ and 4′ positions of ABA are formed upon gate closure and their entrances are located at the interface with PP2Cs. Interestingly, it has been described that chemical compounds interacting with the 3′ tunnel could stabilize gate-closed conformations or even prevent PP2C binding through steric hindrance [79,87,88].

**Figure 5:**
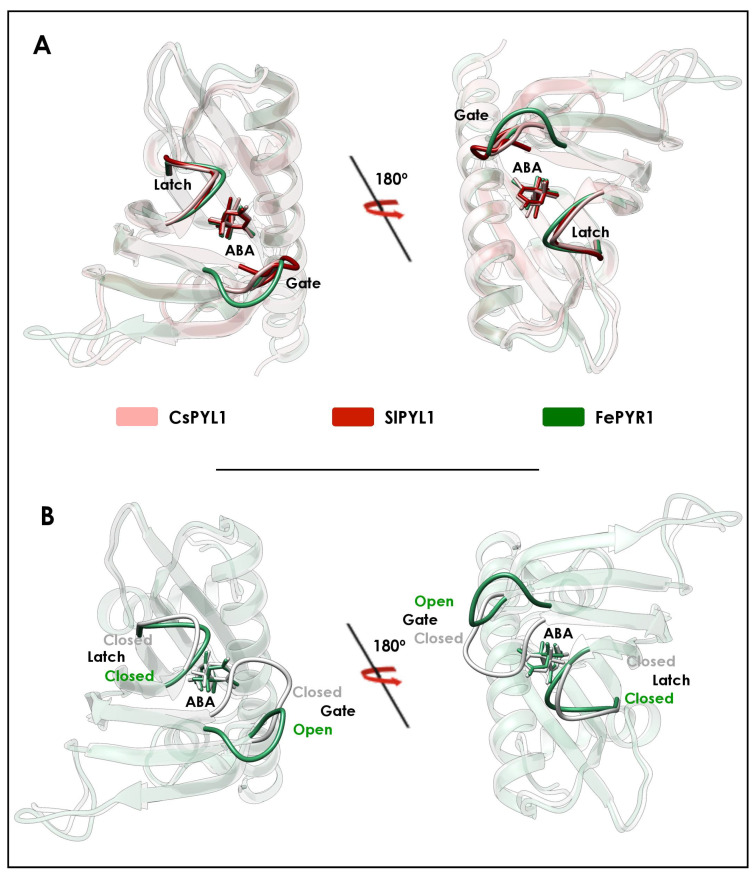
The latch-closed/gate-open ABA-bound intermediate. (**A**) Structural alignment of a latch-closed/gate-open ABA-bound conformation of citrus PYL1 (CsPYL1) (PDB ID: 5MMX), tomato PYL1 (SlPYL1) (PDB ID: 5MOB) and PYR1 from turfgrass (FePYR1) (PDB ID: 5UJV). Latch and gate are highlighted in solid colors. (**B**) Superimposition of the latch-closed/gate-open and latch-closed/gate-closed ABA-bound molecules from FePYR1 crystal structure (PDB ID: 5UJV). Latch and gate are highlighted in solid colors.

**Figure 6:**
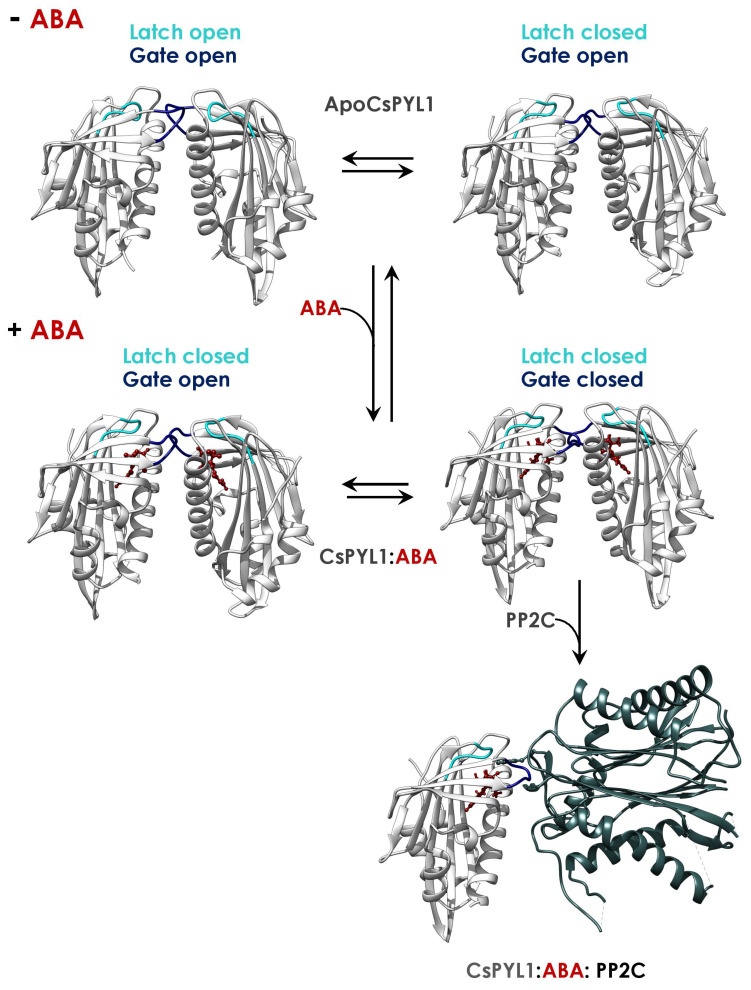
Model of the molecular mechanism for ABA perception. CsPYL1 crystal structures of the apo (PDB ID: 5MMQ), ABA-bound (PDB ID: 5MMX) and ternary complex (PDB ID: 5MN0) forms were used to elaborate this model. Conformational equilibriums of the apo and the ABA-bound forms are represented by horizontal arrows.

Recent structural studies, performed in crops (citrus and tomato), have identified a latch-closed/gate-open ABA-bound intermediate, which may be unable to interact functionally with the PP2C (Figure 3 and Figure 5A) [31]. This intermediate has not been found in any Arabidopsis PYR/PYL structure. In addition, the asymmetric unit of the ABA-bound PYR1 crystal structure from the turfgrass *Festuca elata* contains three ABA-bound monomers forming a crystallographic trimer, in which two monomers are adopting the latch-closed/gate-open intermediate described in citrus and tomato (Figure 5) [77]. Strikingly, the third monomer is adopting the latch-closed/gate-closed conformation (able to interact with PP2C), suggesting the existence of a conformational equilibrium between the latch-closed/gate-open and the latch-closed/gate-closed conformations of ABA-bound receptors. Thus, either the Arabidopsis intermediate is recalcitrant to crystallization, or the mechanism inferred from Arabidopsis crystal structures is slightly different in other plants. In any case, the identification of this intermediate would complement the mechanism deduced from Arabidopsis structures. Taking into account this intermediate, the updated conformational mechanism of ABA perception would be as follows (Figure 6): (1) The apo forms of PYR/PYL proteins are in a conformational equilibrium between a form with the latch and the gate in an open conformation and another form with the latch closed. (2) Then, ABA selects the latch-closed/gate-open conformation and the equilibrium is shifted to another equilibrium between the ABA-bound receptor latch-closed/gate-open and latch-closed/gate-closed forms (Figure 6). (3) The latch-closed/gate-closed ABA-bound conformation of the receptor is selected by the PP2Cs forming the functional ternary complex, which in turn may shift the equilibrium of the ABA-bound receptor forms to the gate-closed/latch-closed form. This strongly suggests that the presence of the PP2C promotes the adoption of the latch-closed/gate-closed PYR/PYL conformation able to interact with the phosphatase, supporting that clade A PP2Cs may be considered as ABA co-receptors [31,82]. Crystal structures of the ternary complexes from the monocot crop *Oryza sativa* also support this model. In the ternary complexes formed by OsPYL2 and OsPYL10, with the phosphatases OsPP2C06 and OsPP2C50, respectively, the receptors are adopting the latch-closed/gate-closed conformation as described in Arabidopsis, citrus and tomato (Figure 4) [44,76]. Hence, from all these data, it is clear that the mechanism of ABA-binding and the consequent formation of the ternary complex with PP2Cs are conserved among crop plants including mono- and eudicotyledons.

Despite PYR/PYL proteins sharing the same general ligand binding mechanism, the biotechnological use of ABA-receptor agonists to improve drought resistance in crops would benefit from a “personalized” and deep structural analysis of each receptor, especially in the ABA-binding site. The reported data on the use of chemical ABA-receptor agonists highlight the importance of the variable sequence regions of the receptors. For example, the synthetic compound pyrabactin acts as an ABA-receptor agonist in all members of Arabidopsis subfamily III except for AtPYL2, for which pyrabactin behaves as an antagonist [26]. Structural and biochemical analysis identified valine 114 in AtPYL2 as the residue responsible for the antagonist conformation of pyrabactin in AtPYL2. In addition, the change of the I137 from AtPYL1 to valine, which is the homolog residue in AtPYL2, converts AtPYL1 from a pyrabactin-activated receptor to a pyrabactin-inhibited receptor [26]. In rice, OsPYL10 exhibits a modest response to pyrabactin and presents a phenylalanine at that position (Phe125 in OsPYL10). Interestingly, changing Phe125 to Ile in OsPYL10 improves pyrabactin in vitro activity by more than 20-fold [76,78]. This is a clear example of how subtle differences in specific residues from a variable region of the ABA binding pocket can dramatically alter the effectiveness of synthetic compounds mimicking ABA. Several examples of the differential responses to the same ABA-receptor agonists between Arabidopsis and crops have been reported. Opabactin is a designed agonist that activates all the wheat PYL members with IC50s in the nanomolar range, including TaPYL8. However, in Arabidopsis, AtPYL8-like subfamily members are not activated by this agonist [54]. Another interesting case involves the PYL8-like members (subfamily I) of date palm (*Phoenix dactylifera*) that are predominantly expressed under abiotic stress conditions [65]. In particular, the PYL8-like receptor Pd27 is activated by quinabactin and cyanabatin, which are ABA-receptor agonists that exclusively activate subfamily III and PYL5 PYR/PYL receptors in vitro in Arabidopsis and tomato [45,55,79]. The elucidation of the crystal structure of wheat and date palm ABA receptors in complex with agonist ligands could identify the cause of this differential behavior and help in the design of potent chemical probes to activate drought resistance. Overall, subtle differences in the ABA-binding pocket might not affect ABA binding, but they are clearly important for the activity of agonist and antagonists.

Structural and biochemical studies of crop ABA-receptors have also shown differences in the oligomerization status of several ABA-receptors compared to Arabidopsis [77,78]. It has been established that members of subfamilies I and II are monomeric and those from subfamily III adopt a dimeric configuration, except for the special case of AtPYL3 that is in an equilibrium dimer-monomer [25]. The oligomerization status of PYR/PYL proteins significantly influences the mechanism of ABA perception because the ABA-induced dissociation of dimeric receptors is required prior to PP2C interaction [29]. Furthermore, dimeric receptors show lower affinity for ABA than monomeric receptors, which directly affects their in vitro PP2C inhibition activity [29,38]. The oligomerization status of the ABA receptors is therefore an important factor that must be considered before identifying the target for ligand design. Among monocots, the case of *Festuca elata* FePYR1 is particularly striking because it is the first example of a PYR1 receptor that exhibits a monomeric state in vitro [77]. Unexpectedly, the ABA binding affinity of FePYR1, as well as its ability to inhibit PP2Cs, is similar to that reported for dimeric AtPYR1, suggesting that the oligomerization status does not affect ABA binding in contrast to previous studies [29]. In addition, studies in rice have shown that OsPYL6 and OsPYL12 adopt a different oligomerization status than their Arabidopsis orthologs AtPYL6 and AtPYL13, respectively [78]. Whereas AtPYL6 and AtPYL13 are monomeric, OsPYL6 is in an equilibrium dimer-monomer and OsPYL12 adopts a dimer configuration. In fact, the authors speculate that OsPYL12 could act as a repressor of ABA signaling by forming heterodimers with other PYR/PYL receptors [78]. It is important to mention that some reports indicate that both AtPYL13 and OsPYL12 do not require ABA to inhibit PP2Cs and consequently they are being considered as ABA-independent receptors. However, the limitations of the in vitro PP2C assays should be taken into consideration to fully evaluate these conclusions. For example, while the natural substrates for clade A PP2Cs are phospho-threonine and phospho-serine peptides, like the activation loop of OST1/SnRK2.6, researchers often use artificial substrates (e.g., *p*-Nitrophenyl phosphate, 4-Methylumbelliferyl phosphate) that could affect the results of the enzymatic assays. The use of natural substrates in PP2C assays or affinity measurements using direct methods like ITC are needed to evaluate the ABA-dependent or ABA-independent nature of these receptors. 

The structural and biochemical analysis of crop ABA receptors confirms the conservation of the ABA perception mechanism among higher plants, which is a reflection of their high sequence similarity. However, the high sequence-structure similarity among ABA receptors cannot explain their specific functions (e.g., AtPYL8) and even less their different responses to designed chemical agonists. Consequently, a structural characterization of crop ABA receptors of biotechnological interest will be required to further understand PYR/PYL biology in crops. These studies will also be essential to optimize the design of synthetic ligands that mimic ABA and to develop new strategies to improve crop yield under drought conditions.

## 6. Concluding Remarks

Food crops are essential to secure food for a growing population. However, our knowledge about PYR/PYL ABA receptors in these plant species is still limited. Further work is required to identify and characterize PYR/PYL receptors in relevant crop species (e.g., sugarcane, cassava and potato). Biochemical data on crop ABA receptors is also very incomplete and would benefit from an extended validation of ABA binding on the new and reported crop ABA receptors. This characterization has already been performed in some crops, like rice and wheat. Interestingly, there are some differences between crop and Arabidopsis receptors, an especially remarkable example being the divergence on their activation by certain ABA-receptor agonists. The gene expression analysis of the different *PYR/PYLs* in crops has also been addressed. Indeed, the down-regulation exerted by ABA on Arabidopsis receptors does not hold in the crop species analyzed in this review, highlighting the difficulty in translating the findings from model systems to crops species. Structural and biophysical approaches using crop PP2Cs would also be valuable to obtain specific and quantitative information on the formation of the ternary complexes, in economically relevant plant species. Furthermore, genetic studies, making use of the latest genome editing technologies available, would be very beneficial to understand the functional role of the different PYL/PYLs in different processes (i.e., stomata closure, root growth, flowering). The recent development of improved transformation methodologies will help in the generation of genome edited crop plants. Genetic, biochemical and structural studies on crop ABA receptors will pave the way to translate PYR/PYL biology into biotechnological solutions able to improve drought resistance in crops.

## Data Availability

Not applicable.
